# Intraductal Carcinoma of the Prostate as a Cause of Prostate Cancer Metastasis: A Molecular Portrait

**DOI:** 10.3390/cancers14030820

**Published:** 2022-02-06

**Authors:** Helen Pantazopoulos, Mame-Kany Diop, Andrée-Anne Grosset, Frédérique Rouleau-Gagné, Afnan Al-Saleh, Teodora Boblea, Dominique Trudel

**Affiliations:** 1 Centre de Recherche du Centre Hospitalier de l’Université de Montréal (CRCHUM), 900 Saint-Denis, Montreal, QC H2X 0A9, Canada; helen.pantazopoulos.chum@ssss.gouv.qc.ca (H.P.); mame-kany.diop.chum@ssss.gouv.qc.ca (M.-K.D.); andree-anne.grosset.chum@ssss.gouv.qc.ca (A.-A.G.); frederique.rouleau-gagne.chum@ssss.gouv.qc.ca (F.R.-G.); afnan.al-saleh.chum@ssss.gouv.qc.ca (A.A.-S.); teodora-b-p@hotmail.fr (T.B.); 2 Institut du Cancer de Montréal, 900 Saint-Denis, Montreal, QC H2X 0A9, Canada; 3 Department of Pathology and Cellular Biology, Université de Montréal, 2900 Boulevard Édouard-Montpetit, Montreal, QC H3T 1J4, Canada; 4 Department of Pathology, Centre Hospitalier de l’Université de Montréal (CHUM), 1051 Sanguinet, Montreal, QC H2X 0C1, Canada

**Keywords:** intraductal carcinoma of the prostate (IDC-P), metastasis, biomarkers, immunohistochemistry, genomic, Raman micro-spectroscopy

## Abstract

**Simple Summary:**

Most men with prostate cancer will live as long as those who do not have prostate cancer. However, some men will die early of their disease due to a particular type of prostate cancer associated with recurrence and metastasis: intraductal carcinoma of the prostate. In this review, we discuss the associations between intraductal carcinoma of the prostate and metastasis, and the contemporary knowledge about the molecular alterations of intraductal carcinoma of the prostate.

**Abstract:**

Intraductal carcinoma of the prostate (IDC-P) is one of the most aggressive types of prostate cancer (PCa). IDC-P is identified in approximately 20% of PCa patients and is associated with recurrence, metastasis, and PCa-specific death. The main feature of this histological variant is the colonization of benign glands by PCa cells. Although IDC-P is a well-recognized independent parameter for metastasis, mechanisms by which IDC-P cells can spread and colonize other tissues are not fully known. In this review, we discuss the molecular portraits of IDC-P determined by immunohistochemistry and genomic approaches and highlight the areas in which more research is needed.

## 1. Introduction

Prostate cancer (PCa) is the most frequently diagnosed cancer of men, accounting for 26% of all malignancies found in North American men, and the second cause of cancer-related death [1]. When PCa is localized, the 5-year survival rate of patients is nearly 100%; yet when PCa has metastasized, the 5-year survival rate steeply declines to 28% despite intensive therapy [2,3]. Many factors are associated with survival in PCa, such as tumour stage and tumour grade. In PCa, the tumour grade is evaluated according to how glands are well formed (pattern 3), unrecognizable (pattern 5) or in between (pattern 4), and added together according to their relative abundance to generate the Gleason score (6 to 10) which is then transformed to grade groups (1 to 5) [4].

**Figure 1:**
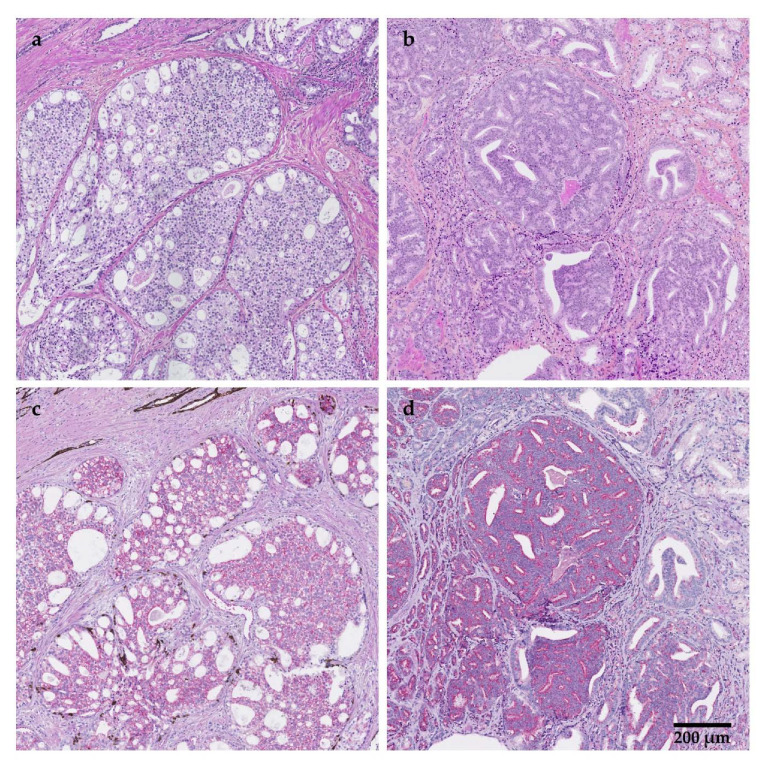
Intraductal carcinoma of the prostate (IDC-P) (**a**,**c**) and cribriform type pattern 4 prostate cancer (CC) (**b**,**d**). Hematoxylin phloxine saffron staining (**a**,**b**) or immunostaining for high molecular weight cytokeratins and p63 (basal cell markers, brown) and α-methylacyl-CoA racemase (prostate cancer marker, red) with hematoxylin and eosin counterstaining (**c**,**d**), highlighting the basal cells surrounding IDC-P and the direct contact between CC and the tumour stroma. Scale bar: 200 μm.

Among the factors influencing PCa survival is intraductal carcinoma of the prostate (IDC-P), a growth of malignant cells found in benign prostatic ducts and acini (Figure 1a,c), recently recognized as a distinct aggressive variant of PCa [5,6,7]. IDC-P is identified in approximately 20% of radical prostatectomies (RPs), often with invasive high-grade, high-risk PCa, and has been associated with early biochemical recurrence, metastasis, castration-resistant prostate cancer (CRPC) and poor survival [8]. Of note, despite similar nomenclature and associations with poor outcomes, IDC-P is morphologically distinct from ductal adenocarcinoma [9]. Briefly, ductal adenocarcinoma is most often directly in contact with the stroma (i.e., is not within a benign duct or acini), and is recognized by columnar pseudostratified epithelium with elongated nuclei [9]. Thus, the two entities should not be confused.

**Figure 2:**
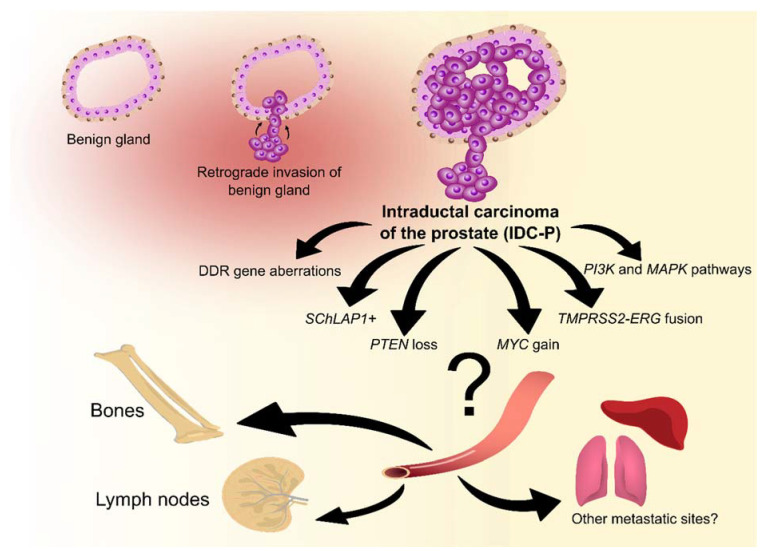
Known molecular alterations for the pathogenesis and progression of intraductal carcinoma of the prostate (IDC-P). DDR: DNA-damage repair; *SChLAP1*: second chromosome locus associated with prostate cancer-1; *PTEN*: phosphatase and tensin homolog; *ERG*: ETS-related gene; PI3K: phosphoinositide 3-kinase; *MAPK*: mitogen-activated protein kinase.

Although the diagnostic criteria and the clinical significance of IDC-P have become clearer since its first description by Kovi et al. in 1985 [7], response to standard treatment is better known, but specific treatment options have yet to be established [10,11,12,13,14,15,16,17,18]. Of all the adverse features associated with IDC-P, metastatic disease is the most threatening to the patient. IDC-P has been associated with lymph node metastasis [19,20,21,22,23,24,25], distant metastasis [26,27,28,29,30,31,32,33,34,35], poor outcome in men with metastatic disease and CRPC [17,18,36,37,38,39,40], PCa-specific death and poor overall survival [25,27,31,36,39,40,41,42,43]. Understanding the molecular cascades underlying the invasion of PCa cells into benign ducts, and their escape from the prostate and from androgen dependency, would help discover biomarkers of aggressive disease and the development of much-needed targeted therapies. Here, we review the associations between IDC-P and metastatic PCa, the current knowledge about treatment response of IDC-P and align these associations with the known molecular alterations of IDC-P (Figure 2) while highlighting areas that could benefit from further investigations.

## 2. IDC-P Is an Independent Parameter for Metastasis and Survival

### 2.1. IDC-P Predicts the Presence of Lymph Node Metastasis at Diagnosis

Lymph node metastasis is associated with shorter disease-free survival in PCa [44]. IDC-P has been frequently associated with lymph node metastasis [19,20,21,22,23,24,25] (summarized in Supplemental Table S1A), with a hazard ratio (HR) of 3.79 to develop lymph node-positive disease, as described in a recent meta-analysis [13]. The first report of this association by O’Brien et al. [19] evaluated the predictive impact of IDC-P in 50 high-risk PCa patients treated with neoadjuvant chemotherapy. Interestingly, only IDC-P (multivariate odds ratio (OR) of 4.6, 95% confidence interval (CI) of 1.5–14.2, *p* = 0.007) could predict the presence of lymph node metastases [19].

In treatment-naïve cohorts, Xu et al. [20] first showed in 316 men with clinically localized, bilateral PCas without metastasis (cT2N0M0) who underwent RP, that IDC-P was associated, but not independently, with lymph node metastasis. Kryvenko et al. [21] compared the clinicopathological characteristics between patients with Gleason score 7 Pca who had lymph node metastases and those without any other metastasis at RP. Of the 368 RP specimens, 184 patients had metastases to lymph nodes, and these patients were significantly more likely to have IDC-P (78/184) than patients that did not have any metastases (38/184) (42.4% vs. 20.7%, *p* < 0.001) [21].

In two retrospective studies in treatment-naïve men, Downes and colleagues [22,23] evaluated IDC-P in conjunction with cribriform type pattern 4 (CC) as a predictive parameter in detecting lymph node metastases. CC is an adverse histopathological feature of the prostate that is now classified as a Gleason pattern 4, and IDC-P and CC are often assessed together due to the difficulty in correctly distinguishing the two histopathologies [45,46,47,48,49] (Figure 1b,d). Accordingly, Downes et al. [22] examined IDC-P and CC together and showed that IDC-P/CC histopathology was present in 94% of all RP specimens from 110 node-positive patients. Although there was no association between the grade at RP and the nodal grade, the grade assigned to the largest node was associated with shorter survival [22]. Downes et al. [23] then evaluated the role of IDC-P/CC in 543 patients who underwent RP, of which 275 patients had matched biopsies. IDC-P/CC was significantly associated with lymph node metastases in the RP and biopsy cohort (multivariate OR: 5.12, 95% CI: 1.37–19.2, *p* = 0.015; and multivariate OR: 4.42, 95% CI: 1.29–15.14, *p* = 0.018, respectively) [23].

Both very small and large-scale studies point to the same associations. Lindberg et al. [24] published a case study in which the DNA alterations in the prostatic ducts colonized by IDC-P shared 83% of the break-point regions with lymph node metastases, directly implicating regional metastatic PCa as an outcome of IDC-P [24]. On the other hand, Dinerman et al. [25] performed a Surveillance, Epidemiology and End Results Program (SEER) retrospective study in which IDC-P alone had predictive value on lymph node metastases in 242 out of 159,777 RP specimens (5.8% vs. 2.4% when IDC-P is absent, *p* < 0.001) [25].

These studies [13,19,20,21,22,23,24,25,31] establish that the presence of IDC-P/CC is strongly associated with lymph node metastasis at diagnosis. However, the only available studies in treatment-naïve men of all grades have been conducted by merging IDC-P with CC. As IDC-P is likely resistant to neoadjuvant therapy with either hormone therapy or chemotherapy [16,19,50,51,52], the role of IDC-P alone as a predictor of lymph node metastasis in patients with higher grade PCa should be better clarified. Interestingly, Hollemans et al. studied 408 men with N0M0 grade group 2 PCas, and observed that the occurrence of lymph node metastasis was not statistically different when comparing men with CC and IDC-P and men with IDC-P alone [53].

### 2.2. IDC-P Predicts the Occurrence of Distant Metastasis

In addition to N1 disease, IDC-P has been often associated with distant metastasis [26,27,28,29,30,31,32,33,34,35] (summarized in Supplemental Table S1B). When comparing 85 men who experienced recurrence detected either by biopsy or radiology, Trinh et al. [26] showed that men with IDC-P at RP developed distant metastasis more frequently than loco-regional recurrence (OR: 6.27, 95% CI: 1.43–27.6, *p* = 0.015). In 206 high-risk PCas, Kimura et al. [31] showed that IDC-P status was significantly correlated with progression-free survival (multivariate HR: 3.07, 95% CI: 1.44–6.58, *p* = 0.0038), which was defined as time from RP to development of local or distant metastases.

In 2019, Tom et al. [27] evaluated the clinical outcomes of IDC-P positive patients and demonstrated that IDC-P was an adverse prognostic parameter of distant metastasis in a cohort of 237 patients with Gleason score 7 PCa treated with external beam radiotherapy. In this cohort, CC was not significantly associated with distant metastasis-free survival unless it was evaluated in conjunction with IDC-P (multivariate HR: 4.18, 95% CI: 1.43–12.28, *p* = 0.01), and IDC-P was not identified without CC in the biopsies [27]. Similarly, van der Kwast et al. [28] studied two cohorts of men with PCas treated by radiation therapy: one including men with intermediate-risk PCas and the other including men with high-risk PCas who were part of a clinical trial evaluating the addition of long-term androgen deprivation therapy to radiation therapy. In this study, IDC-P was associated with shorter time to clinical progression (local, distant or death), when men were treated by radiation therapy only (multivariate HR: 2.33, 95% CI: 1.14–4.76, *p* = 0.02), but only in univariate analysis when men were exposed to androgen deprivation therapy (HR: 2.83, 95% CI: 1.16–6.92, *p* = 0.018) [28].

In an earlier case-control study including 161 men with Gleason score 7 PCas (52 with metastases, disease-specific mortality or both), Kweldam et al. [29] showed that IDC-P was a univariate predictor for distant metastasis. In their study, IDC-P was strongly associated with CC (*p* < 0.001), and both CC and IDC-P were adverse predictors for distant metastasis-free survival. However, IDC-P lost significance in multivariate analyses (multivariate HR for CC: 8.0, 95% CI: 3.0–21, *p* < 0.001; univariate HR for IDC-P: 2.5, 95% CI: 1.2–4.7, *p* = 0.007) [29]. It is worth noting that if CC is highly correlated with IDC-P, including these two factors in a multivariate model may bias the final model.

Other groups have implicated IDC-P/CC together as a predictor of the development of distant metastases [30,32,33,34] in PCa patients. In 2017, Chua et al. [30] evaluated 1325 men treated for localized PCa, and found that patients with IDC-P/CC had an increased likelihood of developing distant metastasis (multivariate HR: 3.31, 95% CI: 1.76–6.21, *p* < 0.001) [30]. In 2019, Hollemans et al. evaluated the impact of IDC-P/CC in 1064 first-line RPs and found that IDC-P/CC predicted shorter metastasis-free survival (multivariate HR: 9.9, 95% CI: 3.9–25.5, *p* < 0.001) [32]. Subgroup analyses showed the same results in the 140 Gleason score 8 RP specimens [33] and in the 854 grade group 1 and 2 RP specimens, although metastases were infrequent in this group [34]. The very few patients who developed distant metastasis (6%) harboured IDC-P/CC in their RPs [34].

Lastly, Isaacsson Velho et al. [35] studied the occurrence of metastasis in 60 men with primary Gleason pattern 5 at RP, of which nine (15%) showed either ductal or intraductal histology. In these men, IDC-P and/or ductal adenocarcinoma were associated with shorter metastasis-free survival (multivariate HR: 3.78, 95% CI: 1.51–9.45, *p* = 0.004) [35].

Globally, regardless of the risk stratification or whether PCa was treated by RP or radiation therapy, IDC-P alone or in combination with CC or ductal adenocarcinoma has been associated with the development of distant metastasis [26,27,28,29,30,31,32,33,34,35]. Interestingly, only Kweldam et al. [29] compared the impact of IDC-P and CC alone and in combination. However, their important study included only Gleason score 7 PCas.

### 2.3. IDC-P Predicts Poor Prognosis in Men with Metastatic PCa

Many groups have assessed the clinical impact of IDC-P in men with metastatic PCas on first diagnosis [17,18,36,37,38,39,40] (summarized in Supplemental Table S1C). One of these groups, led by Zeng, initially verified the time to CRPC in men diagnosed with metastatic disease and IDC-P [36]. Time to CRPC was cut in half when IDC-P was present in the diagnostic biopsy (23 months vs. 46 months, multivariate HR: 4.89, 95% CI: 1.44–16.56, *p* = 0.011). In a second study, Chen et al. [17] evaluated 45 patients initially diagnosed with bone metastatic PCas, which later progressed to CRPC. All patients were subjected to two transperineal prostate biopsies: one at the time of initial diagnosis and a second upon diagnosis of metastatic CRPC. As the prevalence of IDC-P increased from 20% to 62.5% between the two biopsies, IDC-P was associated with shorter time to prostate-specific antigen (PSA) doubling only when taking into account the second biopsy [17]. In another study with the same two-biopsy design, the prevalence of IDC-P increased from 28% to 47% at the time of CRPC, and IDC-P was associated with shorter overall survival (multivariate HR: 1.91, 95% CI 1.11–3.29, *p* = 0.020) [37]. A high amount of IDC-P (>10% of the tumour content) was also associated with shorter CRPC-free survival (multivariate HR: 2.11, 95% CI: 1.52–2.93, *p* < 0.001), and shorter overall survival (multivariate HR: 2.31, 95% CI: 1.38–3.87, *p* = 0.002) in 644 de novo metastatic PCas [38]. Similarly, the same group identified IDC-P as an independent predictor of CRPC-free survival (HR: 1.82, 95% CI: 1.35–2.44, *p* < 0.001) [39].

Other groups also studied the impact of IDC-P in men with de novo metastatic PCa. Kato et al. found that IDC-P was associated with cancer-specific survival (univariate HR: 2.13, 95% CI: 1.14–3.99, *p* = 0.0181) and overall survival (multivariate HR: 2.66, 95% CI: 1.47–4.79, *p* = 0.0012) in a cohort of 150 men with bone metastases at their initial PCa diagnosis [40]. Recently, Abascal-Junquera et al. [18] demonstrated the importance of IDC-P in 118 metastatic CRPC patients as they observed that the time to CRPC was shorter for the 37 men with IDC-P than for the 81 men without IDC-P (10 months vs. 25 months, *p* = 0.007). Furthermore, time to second-line therapy with abiraterone or enzalutamide was also shorter for men with IDC-P (11 months vs. 6 months, *p* = 0.05).

In contrast, Porter and al. [16] assessed overall survival in a small-scale study including 38 men who developed metastases after their initial diagnosis and found no significant difference in survival between men with and without IDC-P at diagnosis, despite a median follow-up of 4.9 years from diagnosis to CRPC [16].

Interestingly, most of these studies [17,18,36,37,38,39,40] establish IDC-P alone, without evaluating the impact of CC, as a prognostic factor in men diagnosed with de novo metastatic PCa to the bones and who progressed to CRPC. It should also be noted that all these studies excluded men with visceral metastases, which, although rare in PCa, could be associated differently with IDC-P.

### 2.4. IDC-P Predicts Death from PCa

The association of IDC-P and poor outcome when diagnosed in M1 PCa is expectedly followed by an association of IDC-P with poor disease-specific survival and overall survival [13,25,27,31,36,37,38,39,40,41,42,43,54] (summarized in Supplemental Table S1D). The effect of IDC-P alone on disease-specific survival has been evaluated in the SEER database [25] in men with localized PCas [43] and in high-risk PCas [31,42]. IDC-P was significantly associated with shorter disease-specific survival in all studies [13,25,27,31,36,37,38,39,40,41,42,43,54] but one [42], with HRs varying from 1.7 to 4.48. Moreover, the association between IDC-P and overall survival has been evaluated in men with de novo metastatic disease (restricted to bone metastases) [36,38,39,40], CRPC [37] and in one cohort of high-risk patients [42], with HRs varying from 1.61 to 2.66. These results are consistent in a recent multivariate analysis [13].

Three studies addressed the combined impact of IDC-P and CC on disease-specific survival [27,29,54]. The first study [29] was the case-control study of men with metastases and/or lethal PCas by Kweldam et al. discussed above, in which IDC-P was associated with distant metastasis only in univariate analysis. Only CC was associated with PCa disease-specific survival (multivariate HR: 5.4, 95% CI: 2.0–15, *p* = 0.001) [29]. The second study included men with Gleason score 7 PCas treated by external beam radiation therapy, and showed that CC alone was not associated with the occurrence of metastasis, but IDC-P combined with CC was associated with metastasis and disease-specific survival (multivariate HR: 14.26, 95% CI: 2.75–74.04, *p* = 0.0016) [27]. Interestingly, in this cohort, IDC-P was not found without CC [27]. In the third study, the effect of IDC-P/CC on overall survival was compared to the effect of non-CC Gleason pattern 4 in the Health Professionals Follow-up Study and the Physicians’ Health Study [54]. The presence of IDC-P/CC was evaluated on tissue microarrays of 0.6 mm-cores built from the primary PCa nodule or the nodule with the highest grade. Elfandy et al. showed that IDC-P/CC was associated with lethal disease (IDC-P/CC: 43/218 lethal cases, non-CC Gleason 4 pattern: 46/600 lethal cases; unadjusted HR: 2.66, 95% CI; 1.75–4.03), but the association lost statistical significance with adjustment for Gleason score, age, body-mass index and cTNM (full model HR: 1.45, 95% CI: 0.92–2.27).

Lastly, in the European Randomized study of Screening for Prostate Cancer (Rotterdam cohort), the incidence of IDC-P/CC in biopsy specimens of 15 men who died of PCa despite having cT1/2 disease with classical Gleason score ≤ 6 were compared to biopsy specimens of 64 men with non-fatal PCas and classical Gleason score ≤ 6. After reclassification following the International Society of Urological Pathology (ISUP) 2014 modified Gleason score, 63% (5/8) of men with grade group two fatal PCas had IDC-P/CC, compared to 13% (2/16) of men with non-fatal, grade group 2 PCas [55].

## 3. Treatment Response

Understanding how IDC-P responds to standard PCa treatment is crucial for the design of new targeted therapies. These questions have mostly been addressed in retrospective studies or in clinical trials with IDC-P as a secondary endpoint [15,17,19,37,50,51,52,56,57,58,59] (summarized in Supplemental Table S2). Currently, two ongoing studies in China focus on IDC-P treatment. One evaluates neoadjuvant androgen-deprivation therapy and abiraterone in IDC-P, and the other evaluates docetaxel and abiraterone in mCRPC and IDC-P. In addition, two other registered studies will be evaluating the presence of IDC-P or CC after neoadjuvant therapy as a secondary endpoint, and another will be evaluating the difference in time-to-recurrence according to IDC-P status as a secondary endpoint after radiation therapy with or without androgen-deprivation therapy. With the growing importance of IDC-P in PCa outcomes, more clinical trials are expected to focus on the treatment of IDC-P.

### 3.1. Response to Neoadjuvant Chemotherapy, Androgen-Deprivation Therapy and Androgen Receptor Axis-Targeted Therapy

The first studies addressing the response to therapy of IDC-P were conducted in the neoadjuvant context in retrospective studies. Efstathiou et al. published [50] a study of 115 men who underwent RP after androgen ablation, alone or in combination with chemotherapy. This series included mostly men with high-grade disease (71% of Gleason score 8–10), and IDC-P/CC were present in 72% of RPs. In multivariate analysis, the presence of IDC-P/CC, margin status and treatment predicted biochemical recurrence (relative risk [RR] for IDC-P/CC: 2.98, standard error: 0.46, *p* = 0.02) [50]. Similarly, O’Brien et al. [19] identified IDC-P in 20% of 50 RPs from men treated with neoadjuvant docetaxel and mitoxantrone for high-risk PCa and linked the presence of IDC-P and CC with shorter time to recurrence (multivariate HR: 2.6, 95%CI: 1.5–4.3, *p* < 0.001 and multivariate HR: 2.3, 95%CI: 1.3–4.0, *p* = 0.003, respectively). In a cohort of men with high-risk PCa treated with neoadjuvant androgen-deprivation therapy, men with IDC-P found at both needle biopsy and RP were also shown to have shorter overall survival than men without IDC-P at needle biopsy (regardless of IDC-P status at RP) or men with IDC-P at needle biopsy but not on RP (multivariate HR: 3.2, 95%CI: 1.47–6.95, *p* = 0.0034) [51].

The presence of IDC-P as a factor of response to neoadjuvant therapy has also been evaluated in post-hoc analysis of clinical trials or as secondary endpoints [52,56,57]. In a pooled analysis of three clinical trials evaluating neoadjuvant abiraterone and/or enzalutamide (androgen receptor axis-targeted therapy) in high-risk and unfavorable intermediate-risk patients, McKay et al. [56] showed that IDC-P was associated with a lack of exceptional pathological response at RP (*n* = 45, 0% vs. 41.7%, *p* = 0.001) and with a lower rate of biochemical-free survival at three years (28% vs. 70%, *p* = 0.004). In the histologic and genomic analyses of RPs from 37 men enrolled in a clinical trial of neoadjuvant androgen-deprivation therapy with enzalutamide [57], IDC-P and nuclear ERG expression were associated with incomplete or nonresponsive cases (univariate analyses, *p* = 0.013 and *p* = 0.002, respectively) but CC was not. Based on a combination of IDC-P and ERG expression with deletion of >50% of chromosome 10 q and loss-of-function or hotspot alterations to *TP53*, a model was constructed to predict complete response after neoadjuvant treatment. This model classified 30/37 cases correctly (area under the curve: 0.89, *p* < 0.0001) [57]. In a trial comparing abiraterone, leuprolide and prednisone with or without apalutamide, as there was no significant difference between treatment arms, IDC-P was associated with higher pathologic T stage (tumour extension), larger residual tumours, increased tumour cellularity and higher residual cancer burden [52].

### 3.2. Response to Chemotherapy or Androgen Receptor Axis-Targeted Therapy

In a study by Chen et al. [17], response to treatment was evaluated in 24 men who were initially diagnosed with bone metastatic PCa before progression to CRPC and who were treated with docetaxel-based chemotherapy. In this cohort, 6/9 men without IDC-P (67%) showed response to chemotherapy while only 3/15 with IDC-P (20%) responded to chemotherapy. The same group compared response to CRPC therapy according to IDC-P status at biopsy (at the time of metastatic CRPC) in a cohort of 96 men [37]. In men without IDC-P, response rates to docetaxel or abiraterone were similar (56% vs. 57%, *p* = 0.70), whereas men with IDC-P showed similar response rates to abiraterone (52%) with only a 22% response rate in the docetaxel group [37].

In 2018, Yamamoto et al. [58] came to the same conclusion after studying a cohort of 79 men diagnosed with metastatic PCa who progressed to mCRPC without local treatment. As all men from this cohort were treated by androgen-deprivation therapy, men who received docetaxel had shorter median survival when their PCa harboured IDC-P (20.5 months vs. 53.2 months, HR: 2.98, 95%CI: 1.02–8.64, *p* = 0.044) [58]. While men who received docetaxel had shorter survival with IDC-P than without IDC-P, men with IDC-P still fared better when they received chemotherapy with a median cancer-specific survival of 14.7 months without chemotherapy (median 20.5 months with chemotherapy, HR: 0.44, 95%CI: 0.22–0.91, *p* = 0.026) [58]. Two years later, the same group compared the response to chemotherapy to the response to abiraterone or enzalutamide in a propensity score matching study including 170 men with CRPC [59]. In each treatment group, IDC-P was associated with shorter overall survival, which was not statistically significant in the abiraterone/enzalutamide group (HR for docetaxel group: 3.08, 95%CI 1.76–5.41, *p* < 0.001; HR for abiraterone/enzalutamide group: 1.63, 95%CI: 0.77–3.47, *p* = 0.19). However, patients with IDC-P did better when they received either abiraterone or enzalutamide compared to docetaxel (HR: 0.48, 95%CI: 0.26–0.86, *p* = 0.01) [59].

### 3.3. Response to Adjuvant Radiation Therapy

Until now, only one study has evaluated the addition of radiation therapy to RPs when IDC-P is present [15]. In this study, the outcomes of 293 men with localized to locally advanced PCa were compared in function of the presence of high-risk factors (grade group 4–5, positive margins, extraprostatic extension or seminal vesicle invasion), which can be treated with adjuvant radiation therapy, and in function of the presence of IDC-P. Globally, men with IDC-P and no high-risk factors had a similar outcome than men without IDC-P but with at least one high-risk factor. In a multivariate analysis for the prediction of biochemical recurrence after RP, IDC-P was associated with shorter time to recurrence (HR: 2.39, 95%CI: 1.44–3.97, *p* = 0.001) and adjuvant radiation therapy protected the men from recurrence (HR: 0.38, 95%CI: 0.17–0.85, *p* = 0.018) [15].

## 4. Molecular Markers of IDC-P

To date, evidence has shown that IDC-P alone or in combination with CC is of tremendous importance to PCa outcome. Understanding the molecular characteristics of IDC-P is crucial for the identification and development of early detection tools and targeted therapies. Our knowledge of IDC-P has grown exponentially since a study in 2000 [60] that first detailed some molecular features of IDC-P in 26 patients, with expression of PSA (specific to prostate tissue), MIB-1 (a proliferation marker) and MUC2 (staining intestinal goblet-cell mucus) mostly in the central area of the proliferation, and androgen receptors (AR) expression mostly at its periphery. Here we describe the known molecular alterations of IDC-P compared to other components of prostatic tissue and PCa, as well as the prognostic impact of the molecular alterations associated with IDC-P.

### 4.1. IDC-P without Associated Invasive Carcinoma

Very few reports of isolated IDC-P have been published [61,62]. Khani et al. characterized four isolated IDC-P cases (three were entirely submitted for histology) as well as 11 cases in which IDC-P was identified in association with low grade (Gleason score 6) PCas [63] (Table 1). From seven sequenced IDC-P lesions (one with isolated IDC-P), four showed activating oncogenic driver mutations involving the *MAPK* and *PI3K* pathways, which are rarely involved in PCa but which are found with high frequency in ductal adenocarcinoma [64]. Moreover, these tumours showed less copy number alterations (CNAs) and a lower percentage of genome alterations. Other identified alterations included phosphatase and tensin homolog (*PTEN*) loss, *CHEK2* and *BRCA2* (DNA-damage repair [DDR] genes) mutations, *CDKN2A, RB1* and *CCND1* (cell cycle genes) mutations, along with other PCa-related alterations such as *MYC* amplification in four cases. Immunohistochemistry (IHC) showed low ETS-related gene (ERG) positivity prevalence (1/15), with partial or complete loss of PTEN in roughly half the cases (7/15), and 56% of discordance for the nine cases with sufficient tissue to evaluate adjacent invasive carcinoma.

### 4.2. IDC-P Compared to High-Grade Intraepithelial Neoplasia (HGPIN) and to Adjacent Invasive Carcinoma

The prostatic ducts can be occupied by many lesions such as high-grade intraepithelial neoplasia (HGPIN), the presumed precursor of PCa, as well as clear cell cribriform hyperplasia and urothelial carcinoma [12]. IDC-P and HGPIN are on a spectrum of cellular atypia, and HGPIN is less atypical than IDC-P. Many studies have attempted to distinguish IDC-P from HGPIN and from lesions with intermediate characteristics, coined “atypical intraductal proliferation” (AIP) [65,66,67,68,69]. Logically, IDC-P has also been compared to adjacent invasive carcinoma. Altogether, the following studies largely demonstrated that IDC-P was distinct from HGPIN but similar to AIP and adjacent invasive carcinoma, at least in terms of ERG and PTEN expression (summarized in Table 2). However, studies comparing IDC-P to HGPIN and adjacent invasive carcinoma using other markers or techniques, such as laser microdissection or spatial transcriptomics, are needed to complete our understanding of the relationships between PCa, IDC and HGPIN.

#### 4.2.1. PTEN and ERG (*TMPRSS2:ERG*)

*PTEN* is a tumour suppressor gene involved in the regulation of the cell cycle and is frequently associated with PCa aggressiveness and metastases [81,82,83,84,85,86,87]. Although *PTEN* loss was initially evaluated through genomic approaches, subsequent studies have shifted toward IHC as the correlation between the two is high, and some tumours presenting *PTEN* inactivation do not show genomic loss [84,88,89]. Similarly, detection of *TMPRSS2:ERG*, a fusion leading to the expression of the oncogene *ERG* in 50% of PCas [90], has moved from fluorescence in situ hybridization (FISH) to IHC [71,91,92]. Contrary to *PTEN*, *TMPRSS2:ERG* fusion or ERG expression has not been clearly linked to PCa outcome and is mostly used as a diagnostic marker [93,94,95,96,97]. Since the expression of ERG and PTEN can be evaluated by IHC, a very accessible histopathology technique, these two markers are the most studied in IDC-P [66,67,68,69,70,71,72,74,75,76,77,78,98].

In a multicentric study in 2007, Mosquera et al. [71] were the first to demonstrate that *TMPRSS2:ERG,* studied with a FISH break-apart probe, strongly correlated with IDC-P (82/87 positive cases) and CC (70/94 positive cases). Of all the morphologic features analyzed in the study, IDC-P had the strongest association with positive *TMPRSS2:ERG* fusion status (RR: 8.312, 95% CI: 2.835–24.371, *p* < 0.001) [71]. Similarly, *ERG* rearrangement was identified in 75% of 48 IDC-P lesions, with 100% of concordance with adjacent carcinoma, while absent in all cases of isolated cribriform HGPIN (0/16 cases) [72]. Interestingly, most *ERG* rearrangements in IDC-P were through deletion, among which 6/36 cases showed duplication of *ERG* rearrangement in combination with deletion of 5′-*ERG*, previously associated with very poor prognosis [98]. Moreover, two cases with *ERG* rearrangement in IDC-P and lymph node metastases, showed that *ERG* status was the same in IDC-P and metastasis but discordant in another tumour foci without IDC-P [72].

It then took a few years before the first description of PTEN and ERG IHC expression in RPs by Lotan et al. in 2014 [66]. Cytoplasmic PTEN expression was lost in 84% (38/45) of IDC-P cases (29/38 showed uniform loss), 100% (15/15) of AIP lesions, but never in HGPIN (0/39). When PTEN was lost in IDC-P, at least a portion of the adjacent invasive carcinoma did not express PTEN in 92% of the cases. In parallel, ERG was expressed in 58% (26/45) of the IDC-P cases, 67% (10/15) of the AIPs and 13% (5/39) of the HGPIN lesions. The concordance levels were 100% between intraductal lesions and the adjacent invasive tumours, but not for HGPIN (of the ERG-positive invasive tumours, only 6% [1/11] were associated with ERG-positive HGPIN). Interestingly, PTEN was lost in 67% of ERG-positive tumours compared to 31% of ERG-negative tumours (*p* = 0.006). In their subsequent biopsy study, similar proportions of expression were observed (PTEN loss in IDC-P: 76%, in AIP: 52%, in HGPIN: 0%; ERG expression in IDC-P: 58%, in AIP: 27%, in HGPIN: 0%) [67]. Lower expression of ERG was found in IDC-P by Schneider and Osunkoya who identified 35% (11/31) of ERG-positive IDC-P cases in a cohort including men who previously received androgen deprivation therapy [75], and by Nie et al. [76] who reported 10% of ERG positive cases in 633 consecutive cases.

In 2017, Shah et al. [68] compared the expression levels of ERG and PTEN in AIP and IDC-P in 106 prostate biopsies. They showed that ERG overexpression was present in 41% of AIP cases and 55% of IDC-P cases, while PTEN loss was observed in 71% and 72% of both lesions, respectively. In contrast, PTEN loss was observed in only 5% of HGPIN lesions and ERG was overexpressed in 16% of HGPIN foci. Concordance of ERG and PTEN expression was above 90% in IDC-P, AIP and adjacent invasive adenocarcinoma. Two years later, the same group evaluated predictors of PTEN loss and found that in their cohort of 260 PCas, IDC-P (HR: 4.993, 95% CI: 3.451–7.223), CC (HR: 2.459, 95% CI: 1.814–3.333) and stromogenic PCa (HR:2.255, 95% CI: 1.634–3.112) were the best predictors of PTEN loss (all *p* < 0.001) [78]. Furthermore, Hickman et al. obtained 100% (46/46) concordance of ERG status between IDC-P/AIP and nearby invasive carcinoma vs. only 7% of HGPIN cases [69]. With PTEN loss in two third of the cases, the concordance between IDC-P and adjacent carcinoma was 81% [69]. PTEN loss has also been observed in almost 90% of 40 IDC-P cases compared to 0% of 40 HGPIN cases [77].

Downes et al. [74] further verified the concordance of ERG and PTEN expression patterns in IDC-P, CC and the adjacent invasive carcinoma in a cohort of 57 PCa cases with cribriform morphology. In this cohort, ERG IHC was concordant between IDC-P, CC and adjacent invasive carcinoma in 98% of the cases (56/57). As expected, in the 44 cases with PTEN loss, 75% had heterogeneous loss. From supplemental data, IDC-P and CC staining was concordant in 86% (either retention or loss) of cases; IDC-P and adjacent acinar carcinoma staining, or CC and adjacent acinar carcinoma staining were concordant in >95% of cases, including heterogeneous loss that was concordant with either retention or loss in the other tumour compartment.

Another concordance study by Haffner et al. [70] addressed the spatial localization of ERG and PTEN status in relationship to PCa. *TMPRSS2:ERG* breakpoints analysis of seven cases showed a common phylogeny between HGPIN, PCa and IDC-P (when present), while *PTEN* loss was subclonal. Therefore, it was suggested that IDC-P represents a late event in PCa.

#### 4.2.2. Multimarker Studies

Beyond *ERG* rearrangements, Bettendorf et al. [73] showed in 2008 that IDC-P was a distinct histopathological lesion from HGPIN, as comparative genomic hybridisation (CGH) revealed chromosomal imbalances in 8/11 IDC-P cases and in 0/10 HGPIN cases, with deletions at 8p, 10q and 13q; however, invasive carcinoma was not studied.

In 2000, mutational studies were also conducted on 20 microdissected RPs to compare loss of heterozygosity (LOH) between HGPIN and IDC-P using 12 polymorphic satellite markers frequently lost in PCa, such as 8p22, 10q23–24 and 21q22.1–22.3 [79]. Briefly, LOH was more frequent in IDC-P (16%) than in Gleason pattern 4 (7%) and HGPIN (2.5%). Of note, when these results were published, CC was most likely evaluated as Gleason pattern 3 [99].

#### 4.2.3. Novel Diagnostic Biomarker: Raman Microspectroscopy

Raman spectroscopy (RS) is a nondestructive imaging technique in which the interaction between photons and tissue is measured based on different molecular bonds producing specific shifts in light wavelength [100]. Using a pen-size hand-held probe, RS can be used in real-time to detect brain tumours [101,102,103] or PCas on gross prostate slices [104] before integration to the da Vinci surgical robot [105]. Raman microspectroscopy (µRS) has also been used to differentiate prostatic cell lines and PCa from benign tissues (reviewed in [100]); however, the technique is too expensive and fastidious for clinical implementation and is meant to analyze frozen tissue, which is unavailable when clinically evaluating the histology of PCa. Accordingly, Grosset et al. [80] developed an economical µRS method that is performed on formalin-fixed paraffin embedded (FFPE) tissues according to standard histopathology protocols, which leads to a diagnosis within 90 min of slide preparation. Using this method, IDC-P was distinguished from invasive PCa with an accuracy, sensitivity and specificity of 95 ± 2%, 96 ± 4%, and 94 ± 2%, respectively in the training cohort. Furthermore, IDC-P could be distinguished from HGPIN with accuracies, sensitivities, and specificities of >95%. Of note, optimization of the machine-learning algorithms improved the ability to differentiate IDC-P from cancer to up to 9% [106].

### 4.3. Cases with IDC-P Compared to Cases without IDC-P

Another important area of IDC-P research is the comparison between cases with and without IDC-P (summarized in Table 3), especially since the aforementioned studies established (mostly with ERG and PTEN expression patterns) that IDC-P was similar to its adjacent invasive carcinoma. However, most of the large-scale genomic studies of PCa were conducted before IDC-P became clinically relevant, and as such, post hoc investigations had to be performed on available frozen sections to identify IDC-P. As the distinction between IDC-P and CC is already not necessarily straightforward on FFPE slides, the low quality of the frozen sections in terms of morphology led most authors to investigate IDC-P and CC together. Furthermore, the study context in which tissues were submitted for sequencing did not permit IHC analysis to distinguish between IDC-P and CC. Therefore, most of our genomic knowledge about the distinction between cases with and without IDC-P are based on studies comparing cases with IDC-P/CC and cases without IDC-P/CC [54,107].

In 277 grade group 2 biopsies, Salles et al. [108] specifically showed that PTEN loss and *MYC* gain were associated with the presence of IDC-P at biopsy (OR: 13.33, 95% CI: 3.85–49.67, *p* < 0.0001) and that CC was larger when both alterations were present. In this study, IDC-P, large CC (>200 µm), as well as combined *MYC* gain and PTEN loss, were associated with non-organ-confined disease at RP. However, in multivariate analysis, only *MYC*/PTEN status remained associated with non-organ-confined disease at RP. Similarly, only *MYC*/PTEN status increased the area under the curve (AUC) of a model predicting non-organ-confined disease using standard prognostic factors. In this cohort, IDC-P, large CC and PTEN loss were associated with shorter time to biochemical recurrence.

In an in silico study, Böttcher et al. [107] showed that the percentage of genome alterations in PCas from men included in The Cancer Genome Atlas Project (TCGA) and in the Canadian Prostate Cancer Genome Network (CPC-GENE) was three-fold higher in men with IDC-P/CC, and included deletions and amplifications in regions previously associated with aggressive PCa, such as 8p deletions (*NKX3.1*), 10q23 deletions *(PTEN*) and 8q amplifications (*MYC*).

In a similar in silico study, Elfandy et al. [54] assessed the alterations associated with IDC-P/CC compared to non-CC Gleason pattern 4 in the TCGA and SU2C/PCF Dream Team cohorts. IDC-P/CC remained significantly associated with deletions at 8p21–22, 6q21, 11q22–23 and 10q23 and gains at 3q11–29 while controlling for percentage of genome alterations and Gleason score. *PTEN* loss was significantly enriched in IDC-P/CC (OR: 1.87, 95% CI: 1.09–3.26, *p* = 0.024). *SPOP* and *ATM* mutations were significantly more frequent in IDC-P/CC than in non-CC Gleason pattern 4, but the frequency of *ERG* fusions was not different between the two groups. Moreover, gene expression profiles of IDC-P/CC showed upregulation of the *mTORC1* and *MYC* pathways and hypermethylation in *CYP26A1*.

Additionally, Olkhov-Mitsel et al. [109] investigated the methylation profile of *ALU, APC, CYP26A1, HOXD3, HOXD8, RASSF1, TBX15, TGF-β* in IDC-P/CC lesions from 91 Gleason score 7 PCas and found an increase in the methylation levels of *APC, RASSF15* and *TBX15* in IDC-P/CC.

Lastly, patients with IDC-P have also been characterized for genomic alterations in a study using liquid biopsies targeting PCa-related genes in a cohort of 245 men with M0 disease, metastatic hormone-sensitive PCas and CRPC [110]. Zhao et al. showed that despite similar mutational rates in the *AR* pathway between men with and without IDC-P, the IDC-P group showed enrichment of alterations of the *AR* negative regulator *NCOR2*. Furthermore, cases with a higher amount of IDC-P harboured more *AR* mutations.

Anecdotally, IDC-P has been shown to sometimes harbour small cell-like changes, which can be shown by expression of thyroid transcription factor (TTF-1) in IDC-P [111,112].

### 4.4. IDC-P and Deleterious Germline and Somatic Alterations of DNA-Damage Repair Genes

Since germline mutations of the *BRCA2* tumour suppressor gene significantly increase the likelihood of developing aggressive PCas [113,114,115,116], many researchers have investigated the link between IDC-P and deleterious germline mutations (Table 4). With the emergence of targeted therapies for men with alterations in DDR genes, histology can help preselect patients who are more likely to benefit from these treatments [117,118,119]. According to the following studies, the National Comprehensive Cancer Network (NCCN) PCa guidelines include IDC-P as an indication to search for mutations in DDR genes [120].

The link between IDC-P and *BRCA2* mutations was first established in 2014 by Risbridger et al. [121]. In their study, they observed high incidence of IDC-P (61%) in 44 patient-derived xenografts (PDXs) from three *BRCA2* carriers, and from one man with a familial history of cancer but without identified *BRCA* mutation (BRCAX), compared to the incidence of IDC-P (8%) in 62 PDXs derived from 12 men with sporadic PCas. Following this observation, they evaluated PCa specimens from 33 *BRCA2* carriers, 62 BRCAX cases and 32 sporadic PCa cases. In this cohort, the incidence of IDC-P was significantly higher in *BRCA2* carriers (42%) compared with sporadic PCa cases (3/32, 9%) (*p* = 0.004). While 26% of BRCAX patients exhibited IDC-P, the incidence was not statistically significant compared with sporadic PCa cases. In men of the *BRCA* and BRCAX groups, the presence of IDC-P was associated with shorter overall survival (HR: 16.9, *p* = 0.0064 and HR: 3.57, *p* = 0.0086, respectively), without any difference between men with IDC-P from the two groups (*p* = 0.35). Of note, because of the low numbers, the effect of IDC-P in men with sporadic PCas could not be compared to the effect of IDC-P in men with *BRCA2*/BRCAX profiles.

In 2017, Taylor et al. [122] associated IDC-P with CNAs portending poor prognosis, such as *BCL6* gain or *MTOR* loss in men with PCas associated with a germline *BRCA2* mutation. In this study, IDC-P and adjacent invasive carcinoma phylogenies were also evaluated in four carriers of germline *BRCA2* mutations and six sporadic PCas. In both groups, IDC-P and the adjacent invasive carcinoma arose from the same clone, leaving no information about the original tumour focus, and *MYC* amplifications were found with high frequencies. However, in the sporadic group, these *MYC* amplifications were present in both tumour compartments in only one case. Furthermore, in the same study, 14 men carrying a deleterious germline mutant *BRCA2* allele were compared to 200 age-matched men with localized PCas to investigate the genomic alterations underlying the aggressiveness of *BRCA2*-mutated PCa. Potential identified drivers of aggressiveness included an amplification of a region of chromosome 3q containing the WNT/β-catenin pathway modulator *MED12L* and an amplification of *MED*, the *MED12L* homolog. These were found in 66% and 44% of the *BCRA2*-mutated cases, respectively. Interestingly, *MED12L/MED12* amplification in PCa, already associated with poor outcomes [126,127], was observed more frequently in IDC-P (75% vs. 17%), and these mutations were not enriched in sporadic tumours with IDC-P [122].

The association between IDC-P and DDR genes has been further investigated in a series of 150 consecutive patients with metastatic or recurrent PCas [123]. IDC-P tended to be more prevalent in men with germline DDR gene alterations compared to men without alterations (24% (5/21) vs. 9% (12/129), *p* = 0.06). The same group then performed an exploratory study and found that from 13 men with pathogenic mismatch-repair (MMR) gene mutations (10 from somatic screening, three from germline screening), three had ductal or IDC-P histologies (23%) compared to 14 out of 114 MMR-proficient men (12%) (*p* = 0.38, Fisher’s exact test) [124].

In a further study of 58 men who were carriers of a germline *BRCA2* mutation matched with 116 non-carriers [125], Lozano et al. observed that the presence of IDC-P features was similar between carriers and non-carriers (36% vs. 50%, *p* = 0.085). However, bi-allelic mutations, or somatic plus germline mutations were found at higher frequencies in men with IDC-P (43% vs. 12%, *p* < 0.001), with similar proportions for men with CC. Of note, in this study, *PTEN* homozygous loss was more prevalent in IDC-P than in CC [125].

Lastly, in the previously mentioned cohort from Zhao et al. that included men with M0 disease, metastatic hormone-sensitive PCas and CRPC [110], patients with and without IDC-P shared a similar germline mutation rate as evaluated by liquid biopsy. However, patients with IDC-P had more frequent germline pathogenic alterations in DDR genes (12% vs. 2%, *p =* 0.024) and homologous recombination genes (11% vs. 2%, *p =* 0.032). Interestingly, the frequencies of DDR mutations increased as PCa without IDC-P progressed from M0 disease to CRPC, but was consistently high in men with IDC-P. All *BRCA2* mutations were identified in men with IDC-P.

### 4.5. IDC-P and Its Impact on PCa Outcome

Besides the above-mentioned study by Risbringer et al. [121], few groups have addressed the molecular features underlying the aggressivity of IDC-P [30,128,129,130] (Table 5). Increasing our knowledge in this field will be central to the development of new therapies for men with IDC-P.

In 2017, Chua et al. [30] evaluated whether IDC-P/CC was associated with hypoxia and genomic instability. In addition to demonstrating an increased risk of metastasis in men with IDC-P/CC (see above), Chua et al. showed that IDC-P/CC tumours harboured a higher percentage of genome alterations (34% vs. 16%, *p =* 0.033), with a nonsignificant increase in the hypoxic tumour subpopulation (64% vs. 45%, *p =* 0.17). In a subsequent study by the same group, a hypoxia signature was significantly associated with IDC-P/CC [128]. Furthermore, Chua et al. [30] showed that men with IDC-P/CC and a high percentage of genome alteration were more likely to develop distant metastasis (HR: 5.5, 95% CI: 2.5–12.2, *p <* 0.0001). Importantly, only one gene was found to be overexpressed in IDC-P/CC-positive tumours: *SChLAP1*, a long non-coding RNA associated with poor outcome in PCa [131,132]. In this cohort [30], men with *SChLAP1* overexpression and IDC-P/CC experienced earlier biochemical recurrence compared to men without IDC-P/CC and without *SChLAP1* overexpression (HR: 2.6, 95% CI: 1.4–4.7, *p =* 0.0027). In a subsequent study, *SCHLAP1:UBE2E3* fusion was highly associated with IDC-P/CC and was found to be enriched in metastases [129].

Very recently, Spieker et al. [130] identified PTEN loss in 86% of IDC-P cases (30/35 RPs) and in 64% of CC lesions (72/112 RPs). In this study, CC was associated with shorter time to biochemical recurrence (multivariate HR: 3.50, 95% CI: 1.89–6.49, *p <* 0.001) but not IDC-P alone. However, combining both was associated with a higher cumulative incidence of biochemical recurrence (multivariate HR: 5.06, 95% CI: 2.21–11.6, *p <* 0.001).

### 4.6. Commercial Classification Assays

An important last question that has been only recently addressed is the association between IDC-P and commercial risk-classification assays (Table 6). We believe this question will become increasingly relevant to the care of men with PCa.

The Oncotype DX Genomic Prostate Score (GPS) assay uses biopsy samples to provide a score ranging from 0 to 100 for men with PCa, to predict the probability of adverse pathology at RP, biochemical recurrence at 3 years and metastasis and PCa-related death at 10 years [135,136,137,138]. In their study including 296 men with a total of 319 prostate biopsies, Greenland et al. [133] showed that among different Gleason pattern 4 tumours, the IDC-P/CC pattern was associated with the highest increase in the GPS, with a mean GPS of 42. Of note, three authors of this study, including the senior author, reported financial interest and/or a relationship with Genomic Health (provider of the Oncotype DX platform).

The Decipher Prostate Cancer Test (GenomeDX Biosciences) is a 22 gene-expression assay that produces a continuous score between 0 and 1 to predict the outcomes of PCa patients at initial prostate biopsy [139,140] and for those who undergo RP [141,142]. This risk stratification method predicts the likelihood of developing metastasis within 5 or 10 years [142,143,144,145,146,147,148,149,150,151,152] and cancer-specific mortality [146,153], thus impacting physician-patient decision making and subsequent patient management [154,155,156,157,158,159]. In a study of 48 men who underwent RP and lymph node dissection for PCa, the Decipher test was used in cases with pT3 disease and/or positive surgical margins and showed that both the presence of IDC-P and CC (OR: 1.92, 95% CI: 0.65–5.67, *p =* 0.24 and OR: 9.60, 95% CI: 1.48–62.16, *p =* 0.02, respectively) increased the Decipher risk classification, although the increase associated with IDC-P was not statistically significant (*n* = 15) [134].

## 5. Conclusions

From the reviewed data, IDC-P is clearly an important factor in the survival of men with PCa and seems to have important effects on response to standard treatments. However, as data about IDC-P are still emerging, organizations have proposed different criteria to diagnose IDC-P in histopathological specimens [160]. The ISUP suggests including IDC-P in the tumour grade with CC [161], whereas the Genitourinary Pathology Society (GUPS) recommends including IDC-P in the tumour volume estimation but excluding it from the tumour grade [10]. While this review was not meant to evaluate grading issues associated with IDC-P, the controversy surrounding the diagnosis of IDC-P highlights the need for more research to characterize the prognostic effect of IDC-P.

Although our molecular knowledge about IDC-P has advanced in recent years, most of it has come from studies that evaluated ERG and PTEN status of IDC-P or from post hoc histological reviews of PCas in large sequencing studies that evaluated the characteristics of IDC-P/CC together. Since its initial description in 1985 by Kovi et al. [7], the research questions that need to be addressed about IDC-P are still numerous and of high clinical relevance.

## Figures and Tables

**Table 1:** Overview of the study [63] describing IDC-P without associated invasive carcinoma in radical prostatectomies.

Method	Type of Alteration	Gene or Chromosome	Mutation/ Alteration	% Positivity (n/n)
NGS	Activating SNV (PI3K/MAPK pathways)	*PIK3CA* *AKT1 MAP2K1* *KRAS* *BRAF*	p.H1047R p.E17K p.I99_K104del p.G13P p.K601E	57% (4/7)
	Other SNV	*PTEN*	Splicing	14% (1/7)
GW CNA analysis	CNA	*PTEN*	Loss	29% (2/7)
IHC	Protein loss	PTEN	–	47% (7/15)
NGS	Other SNV (DNA repair genes)	*BRCA2* *CDK12* *CHEK2*	p.L1740 * p.K756Q, p.K504 * p.I157T	29% (2/7)
	Other SNV	*FOXA1*	p.F266V pF396 *	29% (2/7)
	Other SNV	*SPOP*	p.F133V	14% (1/7)
GW CNA analysis	CNA	*CDKN2A* *RB1* *CCND1*	Loss Loss Gain	71% (5/7)
	CNA	*MYC*	Gain	57% (4/7)
	CNA	*TP53*	Loss	14% (1/7)
	CNA	*CHD1*	Loss	14% (1/7)
	CNA	*TSC2*	Gain	14% (1/7)
	CNA	Chr. 8 (8p)	LOH	43% (3/7)
IHC	Protein overexpression	ERG	–	7% (1/15)

Abbreviations: CNA: copy number alteration; GW: Genome-wide; IHC: immunohistochemistry; LOH: loss of heterozygosity; NGS: next-generation sequencing; SNV: single-nucleotide variant.

**Table 2:** Overview of the studies comparing IDC-P to high-grade intraepithelial neoplasia and to adjacent invasive carcinoma. Only IDC-P results are presented.

Specimen Type	Method	% Positivity (n/n)	Ref.
*TMPRSS2:ERG* fusion			
RP	Sanger sequencing	100% (4/4)	[70]
RP	FISH	94% (82/87)	[71]
RP	FISH (break-apart probe)	75% (36/48)	[72]
*PTEN* loss			
RP	Sanger sequencing	75% (3/4)	[70]
RP	Microsatellite analysis: *PTEN* (10q23) LOH	48% (13/27)	[73]
ERG protein overexpression			
RP	IHC	63% (20/32)	[74]
RP	IHC	61% (28/46)	[69]
RP	IHC	58% (26/45)	[66]
Bx	IHC	58% (29/50)	[67]
Bx	IHC	55% (33/60)	[68]
15 Bx; 8 RP; 6 TURP; 2 RCP	IHC	35% (11/31)	[75]
Bx	IHC	10% (12/128)	[76]
PTEN protein loss			
RP	IHC	89% (32/36)	[77]
RP	IHC	84% (38/45)	[66]
Bx	IHC	76% (38/50)	[67]
RP	IHC	75% (18/24)	[69]
Bx	IHC	75% (61/81)	[78]
Bx	IHC	72% (43/60)	[68]
RP	IHC	72% (23/32)	[74]
Other			
RP	CGH: −1q23 → q32, −5p, −6cen → q22, +7p, +7q, −8p, +8q21.1 → qter, −10p, −10q, −10q21 → qter, −13q, −13q14 → qter, −16q, −16q13 → qter, −17p, −18q, +19p, +19q	73% (8/11)	[73]
RP	Microsatellite analysis: *TP53* (17p13.1) LOH	60% (16/27)	[73]
RP	Microsatellite analysis: *RB1* (13q14.2) LOH	81% (22/27)	[73]
RP	Microsatellite analysis: 3pter–3p24.2, 5q21–22, 6q21–22, 7q31, 8p22, 10q23–24, 11p15.5, 16q23.1–16qter, 18q21, 18q21.33, 21q22.1–22.3 LOH	60% (12/20)	[79]
RP	Raman microspectroscopy	Accuracy, sensitivity, and specificity >85%	[80]

Abbreviations: Bx: biopsy; FISH: fluorescence in situ hybridization; IDC-P: intraductal carcinoma of the prostate; IHC: immunohistochemistry; RCP: radical cystoprostatectomy; RP: radical prostatectomy; TURP: trans-urethral resection of the prostate.

**Table 3:** Overview of the studies comparing cases with IDC-P alone or in combination to cases without IDC-P. For in silico studies, selected results are presented.

N (Cohort)	Specimen	IDC-P alone or with CC	Gene or Alteration	Result	Ref.
266 (TCGA, SU2C/PCF Dream Team)	RP	IDC-P/CC vs. NC4	*PTEN^loss^*	39% vs. 25.5% *p* = 0.024	[54]
			*SPOP^mut^*	17.1% vs. 2.9% *p* < 0.001	
			*ATM^mut^*	7.3% vs. 0.98% *p* = 0.019	
			*EZH2 methylation*	logFC= 0.48, q < 0.001	
			*TIMP2 methylation*	logFC= −0.34, q = 0.01	
			*TIMP3 methylation*	logFC= −0.52, q < 0.001	
			*SLIT2 methylation*	logFC= −0.46, q = 0.01	
260 (TCGA)	RP	IDC-P/CC	Higher PGA: 779 gene deletions	q-value < 0.1	[107]
			Higher PGA: 317 gene amplifications	q-value < 0.1	
88 (TCGA)	RP	IDC-P/CC	*FOXA1^mut^*	15% vs. 5% *p* = 0.007	
			*TP53^mut^*	19% vs. 10% *p* = 0.035	
			*SPOP^mut^*	19% vs. 10% *p* = 0.035	
277	Bx	IDC-P (*n* = 31)	*MYC amplification*	uOR: 2.54 95% CI: 1.10–5.88 *p* = 0.02	[108]
			*PTEN loss*	uOR: 5.01 95% CI: 2.26–11.47 *p* < 0.0001	
			*MYC amplification and PTEN loss*	uOR: 13.33 95% CI: 3.85–49.67 *p* < 0.0001	
91	RP	IDC-P/CC (*n* = 61)	*APC methylation*	Median PMR: 47.3% vs. 31.7% *p* = 0.045	[109]
			*RASSF15 methylation*	Median PMR: 99.2% vs. 69.5% *p* = 0.003	
			*TBX15 methylation*	Median PMR: 21.6% vs. 10.0% *p* = 0.013	
245	Liquid Bx	IDC-P	DDR pathway alterations	11.8% (19/161) vs. 2.4% (2/84) *p* = 0.024	[110]
			HR pathway alterations	11.2% (18/161) vs. 2.4% (2/84) *p* = 0.032	
			*NCOR2 alterations*	21.1% (34/161) vs. 6.0% (5/84) *p* = 0.004	
7	3 RP, 3 Bx, 1 RCP	IDC-P	*TTF-1 overexpression*	100% (3/3)	[110]
1	Bx	IDC-P	*TTF-1 overexpression*	100% (1/1)	[110]

Abbreviations: Bx: biopsy; CC: cribriform type pattern 4; CI: confidence interval, DDR: DNA-damage repair; FC: fold change; HR: homologous repair; IDC-P: intraductal carcinoma of the prostate; NC4: non cribriform Gleason 4; PGA: percent genome altered; PMR: Percent methylation ratio; RCP: radical cystoprostatectomy; RP: radical prostatectomy; TCGA: The Cancer Genome Atlas Project; TTF-1: Thyroid transcription factor-1; uOR: univariate odds ratio.

**Table 4:** Overview of the studies associating IDC-P to deleterious germline and somatic alterations of DNA-damage repair genes.

Specimen	Method	Alteration	Result	Ref.
PDX	Histology review	IDC-P in *BRCA2^mut^* and BRCAX grafts vs. sporadic PCas	61% (27/44) vs. 8% (5/62) *p* = 0.04	[121]
RP	Microdissection and WGS	In *BRCA^mut^* cases *MED12L/MED12* amplification in IDC-P+ vs. −	75% (6/8) vs. 17% (1/6)	[122]
Saliva	NGS	% IDC-P in cases with and without germline mutations of DDR genes	24% (5/21) vs. 9% (12/129) *p* = 0.06	[123]
Saliva	NGS	% IDC-P in cases with germline mutations of MMR genes	23% (3/13 IDC-P+)	[124]
6 Bx, 6 RP, 1 LN (IDC-P = 3)	NGS	*MSH6* germline mutation in IDC-P cases	1/3	[124]
		*MSH6* somatic mutations in IDC-P cases	1/3	
		*TP53* somatic mutations in IDC-P cases	1/3	
		*MSH2* somatic mutation + LOH in IDC-P cases	1/3	
		*MSH6* somatic mutation + LOH in IDC-P cases	1/3	
135 RP, 39 Bx (IDC-P = 79)	FISH	Bi-allelic BRCA2 loss (*LOH + gBRCA2 or bi-allelicDel)*	mOR: 4.3 95% CI: 1.1–16.2 *p* = 0.031	[125]
		*PTEN* homozygous loss	mOR: 5.2 95% CI: 2.1–13.1 *p* < 0.001	

Abbreviations: BRCAX: familial history of prostate cancer without identified BRCA2 mutations; Bx: biopsy; CI: confidence interval; DDR: DNA-damage repair; Del: deletion; FISH: fluorescence in situ hybridization; IDC-P: intraductal carcinoma of the prostate; LN: lymph node; LOH: loss of heterozygosity; MMR: mismatch repair; mOR: multivariate odds ratio; NGS: next-generation sequencing; PCa: prostate cancer; PDX: patient-derived xenograft; RP: radical prostatectomy; WGS: whole-genome sequencing.

**Table 5:** Overview of the studies evaluating the impact of the molecular characteristics of IDC-P alone or in combination on prostate cancer outcome in radical prostatectomies.

N	IDC-P alone or with CC	Method	Measure	Result	Ref.
476	IDC-P/CC	CNA analysis	PGA	34% vs. 16% *p* = 0.033	[30]
156	IDC-P/CC	Microarray analysis	*SCHLAP1* expression	FC 3.23 *p* < 0.001	
393	IDC-P/CC	RNA-ISH	Detection of IDC-P/CC using *SCHLAP1* expression	Accuracy: 82.4% *p* < 0.001	
318	IDC-P/CC	SNP microarray	PGA	*p <* 0.0001	[128]
333 (TCGA) and 215 (CPC-GENE)	IDC-P/CC	Ragnum signature	Hypoxia	*p* < 0.0001	
144	IDC-P/CC	Total RNA-seq	*SCHLAP1:UBE2E3* fusion	FDR:0.0015	[129]
163	IDC-P	IHC	PTEN protein loss	86% (30/35)	[130]
	IDC-P/CC	IHC	BCR cumulative incidence	mHR: 5.06, 95%CI: 2.21–11.6 *p* < 0.001	

Abbreviations: CC: cribriform type pattern 4; CI: confidence interval; CNA: copy number alteration; CPC-GENE: Canadian Prostate Cancer Genome-Network; FC: fold change; IDC-P: intraductal carcinoma of the prostate; IHC: immunohistochemistry; mHR: multivariate hazard ratio; PGA: percent genome altered; RNA-ISH: RNA in situ hybridization; RP: radical prostatectomy; TCGA: The Cancer Genome Atlas Project.

**Table 6:** Overview of the studies evaluating the association between IDC-P and commercial risk-stratification assays.

N	Specimen	IDC-P alone or with CC	Measure	Result	Ref.
319	Bx	IDC-P/CC	Increase in GPS	No Gleason pattern 4: mean GPS = 22.3; IDC-P/CC: mean GPS = 41.8 *p <* 0.001	[133]
48	RP	IDC-P/CC	% cases with high-risk Decipher score	56% vs. 22% *p =* 0.007	[134]
		IDC-P	Increase in Decipher score	OR: 1.92, 95% CI: 0.65–5.67, *p =* 0.24	

Abbreviations: CC: cribriform carcinoma; CI: confidence interval; GPS: Oncotype Dx Genomic Prostate Score^®^; IDC-P: intraductal carcinoma of the prostate; OR: Odds ratio.

## Supplementary Materials

The following are available online at https://www.mdpi.com/article/10.3390/cancers14030820/s1, Table S1: (A) IDC-P predicts the presence of lymph node metastasis at diagnosis; (B) IDC-P predicts the occurrence of distant metastasis. (C) IDC-P predicts poor prognosis in men with metastatic PCa; (D) IDC-P predicts death from prostate cancer; Table S2: Treatment response in men with IDC-P alone or in combination with CC.
