# Supplementary material for: Intraductal Carcinoma of the Prostate as a Cause of Prostate Cancer Metastasis: A Molecular Portrait
Source: Cancers (Basel). 2022 Feb 6;14(3):820. doi: 10.3390/cancers14030820 (PMC8834356; doi:10.3390/cancers14030820)
Supplement: Supplementary file 1 [file cancers-14-00820-s001.zip › cancers-1568954-supplementary.pdf]

cancers

MDPI

Intractal Carcinoma of the Prostate as a Cause of Prostate Cancer Metastasis: A Molecular Portrait

Helen Pantazopoulos, Mame-Kany Diop, Andrée-Anne Grosset, Frédérique Rouleau-Gagné, Afnan Al-Saleh, Teodora Boblea and Dominique Trudel

Table S1. (**A**). IDC-P predicts the presence of lymph node metastasis at diagnosis. (**B**). IDC-P predicts the occurrence of distant metastasis. Italicized lines for studies including the same men, or subgroup of the main cohort. (**C**). IDC-P predicts poor prognosis in men with metastatic PCa. (**D**). IDC-P predicts death from prostate cancer.

(**A**)

First author, reference	Year	N	Specimen type	IDC-P alone or in combination	% of IDC-P in the cohort	Univariate vs multivariate testing	Risk of lymph node metastasis
O'Brien C. [19]	2010	50	50 RP<br>22 Bx	IDC-P	20% (10/50) RP<br>0% (0/22) Bx	Multivariate	OR 4.6, 95% CI: 1.5-14.2, p=0.007
Xu N. [20]	2020	316	RP	IDC-P		Univariate	22.89% (±6.76) vs. 20.73% (±6.56), p=0.043
Kryvenko O.N. [21]	2013	368	RP	IDC-P	32% (116/368)	Univariate	42% (78/184) vs. 21% (38/184), p<0.001
Downes M.R. [22]	2019	110	104 RP<br>6 CP	IDC-P/CC	IDC-P: 65% (71/110)<br>CC: 75% (83/110)	NA	94% (103/110)
Downes M.R. [23]	2021	543	RP	IDC-P/CC	60% (203/340) RP	Univariate	OR: 14.06, 95% CI: 3.32-59.5, p=0.003
Downes M.R. [23]	2021	543	RP	IDC-P/CC	60% (203/340) RP	Multivariate	OR: 5.12, 95% CI: 1.37-19.2, p=0.015
Downes M.R. [23]	2021	275	Bx	IDC-P/CC	10% (20/197) Bx	Univariate	OR: 3.29, 95% CI: 1.28-8.42, p=0.013
Downes M.R. [23]	2021	275	Bx	IDC-P/CC	10% (20/197) Bx	Multivariate	OR: 4.42, 95% CI: 1.29-15.14, p=0.018
Lindberg J. [24]	2015	1	RP	IDC-P	100% (1/1)	NA	IDC-P/metastasis share 83% break-point regions
Dinerman B.F. [25]	2017	159,777 (SEER)	RP	IDC-P	0.002% (242/159,777)	Univariate	5.8% (14/242) vs. 2.4% (3,779/159,535), p<0.001
Hollemans E. [53]	2019	420	RP	IDC-P/CC vs. CC	IDC-P/CC: n=79<br>CC : n=125<br>IDC-P : n=24	Univariate	11.4% vs. 1.6%, p=0.003

Hollemans E. [53]	2019	420	RP	IDC-P/CC vs. IDC-P	IDC-P/CC: n=79<br>CC : n=125<br>IDC-P : n=24	Univariate	p=0.32
Kimura K. [31]	2014	206	RP	IDC-P	50.5% (104/206)	Univariate	24% (25/104) vs. 5% (5/102), p<0.0001

Abbreviations: Bx: biopsy; CI: confidence interval; CC: cribriform carcinoma; CP: cystoprostatectomy; IDC-P: intraductal carcinoma of the prostate; OR: odds ratio; RP: radical prostatectomy; SEER: Surveillance, Epidemiology and End Results Program.

(**B**)

First author, reference	Year	N	Specimen type	IDC-P alone or in combination	% of IDC-P in the cohort	Endpoint	Univariate vs multivariate testing	Other factors	Result
Trinh V.Q. [26]	2018	85	RP	IDC-P	76.5% (65/85)	Distant metastasis at initial recurrence	Multivariate	Age, PSA, % of prostate involved, GG, LVI, positive margins, extraprostatic extension, SVI, and nodal metastasis	OR: 6.27, 95% CI: 1.43–27.6, p=0.015
Kimura K. [31]	2014	206	RP	IDC-P	50.5% (104/206)	PFS (time from RP to local or distant metastases)	Multivariate	Age, clinical T stage, PSA, Bx GS, neoadjuvant and/or adjuvant hormone therapy	HR: 3.07, 95% CI: 1.44–6.58, p=0.0038
Tom M.C. [27]	2019	237 GS7	Bx	IDC-P	13% (30/237)	Distant metastasis-free survival	Multivariate	GG 3, high risk group, 50% or more positive biopsy cores, NCCN risk group, race, PSA, ADT	HR: 4.18, 95% CI 1.43–12.28, p=0.01
Van der Kwast T. [28]	2012	Cohort 1: 118 Cohort 2: 135	1: Bx 2: 95 Bx, 45 TURP	IDC-P	1: 19% (23/118) 2: 22% (30/135)	Clinical PFS	Univariate	–	2: RT + LTAD arm HR: 2.83, 95% CI: 1.16–6.92, p=0.018
Van der Kwast T. [28]	2012	Cohort 1: 118 Cohort 2: 135	1: Bx 2: 95 Bx, 45 TURP	IDC-P	1: 19% (23/118) 2: 22% (30/135)	Clinical PFS	Multivariate	GS	2: RT arm HR: 2.33, 95% CI: 1.14–4.76, p=0.02
Kweldam C.F. [29]	2015	161 GS7	RP	IDC-P	41% (66/161)	Distant metastasis	Univariate	–	HR:2.5, 95% CI: 1.2–4.7, p=0.007
Chua M.L.K. [30]	2017	805	RP	IDC-P/CC	20% (165/805)	Distant metastasis	Univariate	–	p<0.0001
Chua M.L.K. [30]	2017	805	RP	IDC-P/CC	20% (165/805)	Distant metastasis	Multivariate	Clinical T stage, ISUP grade, PSA	HR: 3.31, 95% CI: 1.76–6.21, p<0.001
Hansum T. [32]	*2019*	*1064*	*RP*	*IDC-P/CC*	*53.4% (568/1064)*	*Metastasis-free survival*	*Multivariate*	*PSA, pathologic T stage, GG, margin status, pelvic LN status, single cell/cords, small solid nests, medium to large solid fields, comedonecrosis*	*HR: 9.9, 95% CI: 3.9–25.5, p<0.001*
Hollemans E. [33]	*2021*	*140 GS8*	*RP*	*IDC-P/CC*	*62% (82/140)*	*Metastasis-free survival*	*Univariate*	–	*HR: 6.7, 95% CI: 2.0-21.9), p=0.002*
Hollemans E. [33]	*2021*	*140 GS8*	*RP*	*IDC-P/CC*	*62% (82/140)*	*Metastasis-free survival*	*Multivariate*	*Age, PSA, pathologic T stage, GG, margin status, pelvic LN status*	*HR: 3.5, 95% CI: 1.0-12.3), p=0.05*

Hollemans E. [34]	*2020*	*835 GG1-2*	*RP*	*IDC-P/CC*	*27% (228/835)*	*Distant metastasis*	*NA*	*–*	*6% (13/228) GG2 IDC-P/CC vs 0% (0/399) GG1-2 without IDC-P/CC*
Hollemans E. [34]	*2020*	*835 GG1-2*	*RP*	*IDC-P/CC*	*36% (228/627)*	*LN or distant metastasis*	*NA*	*–*	*10% (22/228) GG2 IDC-P/CC vs 0% (0/399) GG1-2 without IDC-P/CC*
Velho P.I. [35]	2019	60 GG5	RP	IDC-P/ductal	15% (9/60)	Metastasis-free survival	Univariate	–	HR: 3.15, 95% CI: 1.3-7.6, p=0.011
Velho P.I. [35]	2019	60 GG5	RP	IDC-P/ductal	15% (9/60)	Metastasis-free survival	Multivariate	Age, PSA, SVI	HR: 3.78, 95% CI: 1.51-9.45, p=0.004

Abbreviations: ADT: androgen deprivation therapy; Bx: biopsy; CI: confidence interval; CC: cribriform carcinoma; GG; grade group; GS: Gleason score; HR: hazard ratio; IDC-P: intraductal carcinoma of the prostate; LN: lymph node; LTAD: long-term androgen deprivation; LVI: lymphovascular invasion; OR: odds ratio; PFS: progression-free survival; NCCN: National Comprehensive Cancer Network®; PSA: prostate specific antigen; RP: radical prostatectomy; RT: radiotherapy; SVI: seminal vesicle invasion; TURP: transurethral resection of the prostate.

(**C**)

First author, reference	Year	N	Specimen type	IDC-P alone or in combination	% of IDC-P in the cohort	Endpoint	Univariate vs multivariate testing	Other factors	Results
Chen Z. [17]	2015	45	Bx	IDC-P	62.5% (28/45)	PSA doubling time < 30 days	Univariate	–	46.4% (13/28) vs. 5.9% (1/17), p=0.004
Zhao T. [37]	2017	131	Bx	IDC-P	47.3% (62/131)	PSA doubling time < 30 days	Univariate	–	37.1% (23/62) vs. 18.8% (13/69), p=0.019
Chen Z. [17]	2015	45	Bx	IDC-P	62.5% (28/45)	PSA Progression	Univariate	–	331.4 ± 18.9 vs. 65.8 ± 7.6, p=0.009
Zhao T. [36]	2015	278	Bx	IDC-P	20.5% (57/278)	CRPC-free survival	Univariate	–	46.05 ± 1.39 vs. 22.98 ± 1.80 mo, p<0.001
Zhao T. [36]	2015	278	Bx	IDC-P	20.5% (57/278)	CRPC-free survival	Multivariate	GS, ALP, Hg, PSA	46.05 ± 1.39 vs. 22.98 ± 1.80 mo, HR = 4.886, 95% CI: 1.444–16.555, p=0.011
Zhao J. [38]	2019	644	Bx	IDC-P	28.0% (180/644)	CRPC-free survival	Multivariate	GS, ECOG score, ALP, LDH, Hg, IDC-P proportion, IDC-P pattern, IDC-P based risk group	HR: 1.63, 95% CI 1.24–2.14, p<0.001
Zhao J. [38]	2019	644	Bx	>10% IDC-P (tumour content)	12.6% (81/644)	CRPC-free survival	Multivariate	GS, ECOG score, ALP, LDH, Hg, IDC-P pattern, IDC-P based risk group	HR: 2.11, 95% CI: 1.52–2.93, p<0.001
Zhao J. [39]	2018	314 (training cohort)	Bx	IDC-P	27.1% (85/314)	CRPC-free survival	Multivariate	Age, GS, ECOG score, PSA, Hg, ALP	HR: 1.82, 95% CI: 1.35–2.44, p<0.001
Abascal-Junquera J.M. [18]	2021	118	Bx	IDC-P	31.4% (37/118)	CRPC-free survival	Univariate	–	24.7 mo (IQR 16.7–32.7) vs. 10.2 mo (IQR 4.2–16.2), p=0.007
Abascal-Junquera J.M. [18]	2021	118	Bx	IDC-P	31.4% (37/118)	Time to second-line therapy	Univariate	–	6.2 mo (IQR 3.2–9.2) vs. 10.6 mo (IQR 5.6–15.6), p=0.05

Abbreviations: ALP: alkaline phosphatase; Bx: biopsy; CFS: castrate-resistant prostate cancer-free survival; CI: confidence interval; CRPC: castrate-resistant prostate cancer; ECOG: Eastern Cooperative Oncology Group; GS: Gleason score; Hg: Hemoglobin; HR: hazard ratio; IDC-P: intraductal carcinoma of the prostate; LDH: lactate dehydrogenase; mo: months; PSA: prostate specific antigen; RP: radical prostatectomy; TURP: transurethral resection of the prostate.

(**D**)

First author, reference	Year	N	Specimen type	IDC-P alone or in combination	% of IDC-P in the cohort	Univariate vs multivariate testing	Other factors	Results
Overall survival								
Kimura K. [31]	2014	206	RP	IDC-P	50.5% (104/206)	Univariate	–	p<0.0001
Zhao T. [36]	2015	278	Bx	IDC-P	20.5% (57/278)	Multivariate	GS, ALP, Hg, PSA	50.38 ± 1.18 mo vs. 36.43 ± 2.10 mo, HR = 1.945, 95% CI: 1.111–3.407, p=0.020
Abascal-Junquera J.M. [18]	2021	118	Bx	IDC-P	31.4% (37/118)	Univariate	–	38 mo (CI 95% 19.9–48.06) vs. 57.9 mo (CI 95% 56.4–59.5), p=0.001
Porter L.H. [16]	2018	38	Bx, RP, TURP	IDC-P	63% (24/38)	Multivariate	Age	3.2 years vs 1.8 years, HR: 0.75, p=0.47
Zhao T. [37]	2017	131	Bx	IDC-P	47.3% (62/131)	Multivariate	CFS, GS, therapeutic scheme, ECOG score, ALP, LDH	HR: 1.91, 95% CI 1.11-3.29, p=0.020
Zhao J. [38]	2019	644	Bx	IDC-P	28.0% (180/644)	Multivariate	GS, ECOG score, ALP, LDH, Hg, IDC-P pattern, IDC-P based risk group	HR: 1.80, 95% CI 1.14–2.82, p=0.011
Zhao J. [38]	2019	644	Bx	>10% IDC-P (tumour content)	12.6% (81/644)	Multivariate	GS, ECOG score, ALP, LDH, Hg, IDC-P pattern, IDC-P based risk group	HR: 2.31, 95% CI: 1.38–3.87, p=0.002
Zhao J. [39]	2018	314 (training cohort)	Bx	IDC-P	27.1% (85/314)	Multivariate	Age, GS, ECOG score, PSA, Hg, ALP	HR: 1.61, 95% CI: 1.09–2.40, p=0.017
Kato M. [40]	2016	150	Bx	IDC-P	67% (100/150)	Multivariate	PSA, cT stage, GS, visceral metastasis, Gleason pattern 5, performance status, pain, Hg, ALP	HR: 2.66, 95% CI: 1.47–4.79, p=0.0012
Disease-specific survival								
Elfandy H. [54]	2019	818 (HPFD/PHS cohorts)	RP	IDC-P/CC vs. NC4	27% (218/818)	N/A	–	IDC-P/CC: 19.7% (43/218) lethal cases, non-CC Gleason 4 pattern: 7.7% (46/600) lethal cases
Elfandy H. [54]	2019	818 (HPFD/PHS cohorts)	RP	IDC-P/CC vs. NC4	27% (218/818)	Multivariate	GS	HR: 1.62; 95% CI: 1.05-2.49
Elfandy H. [54]	2019	818 (HPFD/PHS cohorts)	RP	IDC-P/CC vs. NC4	27% (218/818)	Multivariate	GS, age, BMI	HR: 1.67; 95% CI: 1.08-2.59

Elfandy H. [54]	2019	818 (HPFD/PHS cohorts)	RP	IDC-P/CC vs. NC4	27% (218/818)	Multivariate	GS, age, BMI, cTNM	HR: 1.45; 95% CI: 0.92-2.27
Kato M. [42]	2018	204	Bx	IDC-P	36% (74/204)	Univariate	–	5-year: 23.9% vs. 3.2% 10-year: 33.7% vs. 9.0% 15-year: 52.7% vs. 23.7%, p<0.0001
Kato M. [42]	2018	204	Bx	IDC-P	36% (74/204)	Univariate	–	HR: 2.624, 95% CI: 1.117-6.164, p=0.0268
Saeter T. [43]	2017	283	Bx	IDC-P	35% (98/283)	Multivariate	PSA, clinical T stage, curative treatment	HR: 2.6, 95% CI: 1.5–4.3, p=0.001
Saeter T. [43]	2017	283	Bx	IDC-P	35% (98/283)	Multivariate	PSA, clinical T stage, curative treatment, and GG	HR: 1.7, 95% CI: 0.9–2.9, p=0.07
Dinerman B.F. [25]	2017	159,777 (SEER)	RP	IDC-P	0.002% (242/159,777)	Multivariate	Pathologic T stage, GS, LN status	HR:3.0, 95% CI:1.5-5.7, p<0.01
Kimura K. [31]	2014	206	RP	IDC-P	50.5% (104/206)	Multivariate	Age, clinical T stage, PSA, Bx GS, neoadjuvant and/or adjuvant hormone therapy	HR: 4.48, 95% CI:1.22-16.41, p=0.0238
Tom M.C. [27]	2019	237	Bx	IDC-P	13% (30/237)	Multivariate	GG 3, high risk group, ≥50% positive Bx cores, NCCN risk group, race, PSA, ADT	HR: 14.26, 95% CI 2.75–74.04, p=0.0016
Kweldam C.F. [29]	2015	161 GS7	RP	IDC-P	41% (66/161)	Univariate	–	HR:2.3, 95% CI: 1.1–4.7, p=0.028
Kweldam C.F. [29]	2015	161 GS7	RP	IDC-P	41% (66/161)	Multivariate	Age, PSA, GS, pathologic T stage, surgical margin, positive LN, Gleason 4 patterns, tertiary Gleason 5	HR: 1.0, 95% CI: 0.40–2.7, p=0.93
Kato M. [40]	2016	150	Bx	IDC-P	67% (100/150)	Univariate	–	HR: 2.13, 95% CI: 1.14–3.99, p=0.0181
Alberts A.R.[55]		24 cGS ≤6 men reclassified to the ISUP 2014 mGS 3+4=7	Bx	IDC-P/CC	29.2% (7/24)	NA	–	IDC-P/CC: 63% (5/8) of GG 2 fatal PCas had IDC-P/CC vs. 13% (2/16) of non-fatal GG 2 PCas

Abbreviations: ADT: androgen deprivation therapy; ALP: alkaline phosphatase; BMI: body mass index; Bx: biopsy; CFS: castrate-resistant prostate cancer-free survival; cGS: classical Gleason Score; CI: confidence interval; CRPC: castrate-resistant prostate cancer; ECOG: Eastern Cooperative Oncology Group; GG; grade group; GS: Gleason score; Hg: Hemoglobin; HPFS: Health Professionals Follow-up Study; HR: hazard ratio; IDC-P: intraductal carcinoma of the prostate; LDH: lactate dehydrogenase; NCCN: National Comprehensive Cancer Network®; mGS: modified Gleason Score; NC4: non-cribriform Gleason 4; mo: months; PHS: Physicians' Health Study; PSA: prostate specific antigen; RP: radical prostatectomy; SEER: Surveillance, Epidemiology and End Results Program, TURP: transurethral resection of the prostate.

Table S2. Treatment response in men with IDC-P alone or in combination with CC.

First author	Year	# of patients	Treatment group	IDC-P alone or in combination	% of positivity	Endpoint	Univariate or multivariate	Other factors	Risk measure
Response to neoadjuvant chemotherapy, androgen-deprivation therapy or androgen receptor axis-targeted therapy									
Efstathiou E. [50]	2010	115	Neoadj ADT±CHT	IDC-P/CC at RP	72% (83/115)	BCR	Multivariate	Margin status, treatment group	If IDC-P+, RR: 2.98, standard error: 0.46, p=0.02
O'Brien C [19]	2010	50	Neoadj CHT	IDC-P at RP	20% (10/50)	BCR	Multivariate	Bx GS, cT stage; PSA	HR: 2.6, 95%CI 1.5-4.3, p<0.001
Kato M [51]	2020	145	Neoadj ADT	IDC-P at Bx and RP (+/+) vs (Bx-/RP± or Bx+/RP-)	26.2% (38/145)	Overall survival	Multivariate	GS, Gleason pattern 5, n of + core at Bx, tumour volume at Bx, cT stage, PSA	HR: 3.2, 95%CI: 1.47-6.95, p=0.0034)
McKay R [56]	2021	117	Neoadj abiraterone and/or enzalutamide	IDC-P at RP	46.7% (21/45)	Complete pathologic response	Univariate	–	0% vs. 41.7%, p=0.001
McKay R [56]	2021	117	Neoadj abiraterone and/or enzalutamide	IDC-P at RP	46.7% (21/45)	3-years BCR-free rare	Univariate	–	IDC-P+ 27.6%, 95%CI: 8.9-50.3 vs. IDC-P- 70.4%, 95% CI 47.7-84.7, p=0.004
Wilkinson S [57]	2021	37	Neoadj ADT+enzalutamide	IDC-P	29.7% (11/37)	Pathologic response	Univariate	–	9.1% vs 53.8%, p=0.013
McKay R [52]	2021	100	Neoadj abiraterone, leuprolide and prednisone ± apalutamide	IDC-P	34% (34/100)	Pathologic response	Univariate	–	Larger tumor, more LN metastasis regardless of treatment arm
Response to chemotherapy or androgen receptor axis-targeted therapy									
Chen Z [17]	2015	24	CHT for CRPC	IDC-P	62.5% (15/24)	PSA response	Univariate	–	20% vs 67%, p=0.022
Zhao J [37]	2017	96	CHT or abiraterone for CRPC	IDC-P	47.3% (62/131)	PSA response	Univariate	–	In IDC-P+: abiraterone: 52.4% vs. docetaxel: 21.7%, p=0.035
Yamamoto A [58]	2018	79	ADT ± docetaxel for CRPC	IDC-P	78.5% (62/79)	Cancer-specific survival	Univariate	–	IDC-P+, with CHT: 20.5 mo vs. IDC-P - 53.2 mo, HR: 2.98, 95%CI: 1.02-8.64, p=0.044
Yamamoto A [58]	2018	79	ADT ± docetaxel for CRPC	IDC-P	78.5% (62/79)	Cancer-specific survival	Univariate	–	IDC-P+, with CHT: 20.5 mo vs no CHT: 14.7 mo, HR: 0.44, 95%CI: 0.22-0.91, p=0.026

First author	Year	# of patients	Treatment group	IDC-P alone or in combination	% of positivity	Endpoint	Univariate or multivariate	Other factors	Risk measure
Yamamoto A [59]	2020	170	CHT vs. abiraterone or enzalutamide	IDC-P	60.6% (103/170)	Overall survival	Univariate	--	abiraterone/ enzalutamide vs CHT in IDC-P+: HR 0.48, 95% CI 0.26–0.86; p=0.01
Response to adjuvant radiation therapy									
Trinh VQ [15]	2019	293	RP ± adjuvant radiation therapy	IDC-P	24.9% (73/293)	Time to BCR	Multivariate	Grade, stage, margin status, IDC-P	Adjuvant radiation therapy, HR: 0.38, 95%CI 0.17-0.85, p=0.018

Abbreviations: ADT: androgen-deprivation therapy; BCR: biochemical recurrence; Bx: biopsy; CC: cribriform carcinoma; CHT: chemotherapy; CI: confidence interval; CRPC: castrate-resistant prostate cancer; GS: Gleason score; IDC-P: intraductal carcinoma of the prostate; HR: hazard ratio; LN: lymph node; mo: months; Neoadj: neoadjuvant; PSA: prostate specific antigen; RP: radical prostatectomy; RR: relative risk.
